# Signals of inequity in the care pathway: rural lab diagnosis, systemic therapy delays, and RAS mutation differences in metastatic colorectal cancer

**DOI:** 10.3389/fonc.2026.1826846

**Published:** 2026-06-02

**Authors:** Adam Ismail, Amandri Dahanayake, Shahid Ahmed, Yagan Pillay, Areej Khatib

**Affiliations:** 1 College of Arts and Sciences, University of Saskatchewan, Saskatoon, SK, Canada; 2 Department of Medical Oncology, College of Medicine, University of Saskatchewan, Saskatoon, SK, Canada; 3 Division of General Surgery, College of Medicine, University of Saskatchewan, Prince Albert, SK, Canada; 4 Department of Pathology and Laboratory Medicine, College of Medicine, University of Saskatchewan, Prince Albert, SK, Canada

**Keywords:** access to oncological services, care pathway analysis, diagnostic lab location, metastatic CRC (mCRC), RAS biomarker testing, treatment delay

## Abstract

**Introduction:**

Colorectal cancer (CRC) remains a leading cause of mortality in Canada, with outcomes often influenced by geographic disparities. While biomarker testing for RAS, BRAF, and microsatellite instability (MSI) is now standard for metastatic CRC (mCRC), it is unclear if these diagnostic advances are equitably accessible to rural populations.

**Methods:**

We conducted a retrospective, population-based cohort study of 818 patients diagnosed with stage IV CRC in Saskatchewan between 2017 and 2022. We evaluated differences based on patient residence (rural vs. urban) and diagnosing laboratory location (rural vs. urban) regarding clinical characteristics, biomarker status, treatment delays, and overall survival.

**Results:**

Rural patients had higher odds of right-sided tumors (aOR 1.76) and rectal tumors (aOR 1.52) compared to urban residents. Notably, RAS mutations were more frequently identified in rural patients (11.2% vs. 1.6%, p=0.021). While overall survival did not differ significantly by residency (median 250 vs. 270 days, p=0.774), patients diagnosed in rural laboratories experienced significantly longer delays from diagnosis to the initiation of chemotherapy (66.5 vs. 55 days, p=0.011) and a clinically relevant, though not statistically significant, delay in immunotherapy (77.5 vs. 65 days). Multivariable analysis confirmed that older age and poorer performance status were the primary predictors of mortality, rather than geography.

**Discussion:**

In Saskatchewan, stage IV CRC survival is comparable between rural and urban patients; however, the care pathway is hindered by systemic therapy delays linked to rural diagnostic laboratory locations. While survival rates are currently similar, the delays associated with rural laboratory workups represent an actionable target for improvement.

## 1 Introduction

Colorectal cancer (CRC) is the third most commonly diagnosed malignancy worldwide and the second leading cause of cancer-related mortality in Canada (1). Approximately 6.25% of individuals in Canada are expected to develop CRC during their lifetime, and CRC consistently ranks as the third most prevalent cancer across all Canadian provinces (1). Geographic location has been proposed as a determinant of CRC outcomes, although the direction and magnitude of rural-urban disparities remain inconsistent across the literature. For example, an Alberta population-based study of stage II-III colon cancer reported significantly lower overall survival among rural compared with urban patients (2), whereas a nationwide Canadian study found no association between rural residence and CRC stage at diagnosis (3). On an international level, Australian data demonstrate longer diagnostic and treatment delays for rural CRC patients (4), while a recent US systematic review identified rural-urban disparities across screening, staging, and treatment (5). Collectively, these findings suggest that rural-urban differences in CRC outcomes are context-dependent and influenced by regional healthcare structures, disease stage, and outcome metrics. Tumor stage remains the main prognostic determinant in CRC (6).

In Canada, approximately half of CRC cases are diagnosed at stage III or IV (3). Survival declines steeply with increasing stage: five-year net survival for colon and rectal cancer decreases by approximately 60% when diagnosed at stage IV compared with stage III (7). Stage IV (metastatic) CRC carries a particularly poor prognosis, with reported five-year net survival rates of 11% for colon cancer and 13% for rectal cancer, compared with an all-stage net survival of 67%1. Beyond stage, molecular biomarkers, including microsatellite instability (MSI) status and mutations in BRAF and RAS, have demonstrated independent associations with prognosis and therapeutic response (8). Consequently, timely biomarker testing has become central to treatment selection and survival optimization in mCRC.

Several studies have examined outcomes for patients of mCRC in Saskatchewan. An analysis of patients diagnosed between 2009 and 2013 found no difference in access to first-line systemic therapy or overall survival based on travel distance to a cancer center, suggesting equitable initial treatment delivery across the province (9). However, a subsequent study of patients diagnosed between 2006 and 2010 demonstrated that shorter travel distance (<100 km) was associated with a significantly higher likelihood of receiving second- or later-line systemic therapy (10). Additional Saskatchewan studies evaluating cohorts diagnosed between 1992 and 2005 reported improved survival among stage IV CRC patients who underwent surgical resection of the primary tumor, independent of other prognostic variables (6, 11, 12). Chemotherapy, metastectomy, and receipt of subsequent lines of therapy were also associated with improved survival, whereas older age, poor performance status, and high-grade tumors predicted worse outcomes (11).

To our knowledge, all prior Saskatchewan studies of stage IV CRC pre-date the routine implementation of contemporary biomarker testing and the widespread adoption of novel systemic therapies. Since 2018, provincial guidelines have recommended universal mismatch repair (MMR) testing for all CRC patients, with extended RAS and BRAF testing for those with metastatic disease to inform first-line treatment decisions (13). These advances have enabled biomarker-driven treatment strategies, including immune checkpoint inhibition for dMMR tumors and anti-EGFR therapy for RAS wild-type disease (13).

Whether these diagnostic and therapeutic innovations have been equitably implemented across Saskatchewan, particularly between rural and urban regions, has not yet been systematically evaluated.

In Saskatchewan, anatomical pathology services are centralized within two urban-based laboratories and supported by two rural-based laboratories. Limited local infrastructure in rural settings—and the need to refer biomarker testing to urban centers—raises concerns about potential inequities in diagnostic timeliness and completeness, particularly given the province’s large geography and complex specimen transport pathways. Similar service models exist across other Canadian provinces and internationally, where centralized testing may create comparable rural–urban gaps.

In this study, we report outcomes from a contemporary, population-based cohort of patients diagnosed with stage IV colorectal cancer (CRC) in Saskatchewan between 2017 and 2022. Our objectives were to evaluate rural–urban differences using two complementary definitions: patient residence (rural versus urban) and the location of the diagnosing pathology laboratory (rural versus urban). We compared clinical and molecular characteristics, biomarker testing patterns, treatment utilization and delays, and overall survival within contemporary, biomarker-informed mCRC care.

## 2 Methods

### 2.1 Study design and data sources

We conducted a retrospective cohort study of patients diagnosed with stage IV colorectal cancer in Saskatchewan between January 1, 2017, and December 31, 2022. Data were obtained from the Saskatchewan Cancer Agency (SCA) and the provincial electronic cancer registry. The SCA is responsible for coordinating cancer care across the province, operating two tertiary cancer centers, namely the Allan Blair Cancer Center in Regina and the Saskatoon Cancer Centre, delivering oncology services through 16 regional hospitals via the Community Oncology Program of Saskatchewan. The provincial cancer registry systematically captures all incident cancer diagnoses in Saskatchewan2.

### 2.2 Study population

A total of 838 cases of stage IV CRC were screened. After excluding duplicates, non-adenocarcinoma pathologies, and records with incomplete data, a final cohort of 818 patients was analyzed. Eligible cases included all primary colorectal adenocarcinomas coded as ICD-O-3 C18–C20. Operational approval was obtained from the Saskatchewan cancer agency.

### 2.3 Urban-rural classification

Geography was classified according to the Statistics Canada 2021 Population Centre (POPCTR) framework. Under this definition, municipalities are categorized into three groups based on population size: small population centers (1,000–29,999), medium population centers (30,000–99,999), and large urban population centers (100,000 or more) 3. For the purposes of this study, “Urban” residents were defined as those living in large urban population centers, which in Saskatchewan include only the cities of Saskatoon and Regina. “Rural” residents were defined by combining small and medium population centers. This binary classification was chosen because medium-sized centers in Saskatchewan often rely on the same centralized tertiary oncology referral pathways as smaller rural communities, distinguishing them from the province’s two major urban hubs. Under this classification, approximately half of Saskatchewan’s population is considered rural. Patient residence at the time of diagnosis was determined using postal code data linked to the provincial registry.

### 2.4 Statistical analysis

Descriptive statistics were used to summarize baseline demographic, clinical, biomarker, and treatment characteristics by rural versus urban residence. Categorical variables were evaluated using chi-square or Fisher’s exact tests, and continuous variables, including treatment intervals, were compared using Mann-Whitney U tests. To account for the small number of observed events in mutation subgroups, Firth penalized logistic regression was utilized for multivariable biomarker analysis. Survival outcomes were estimated using the Kaplan-Meier method, and independent predictors of mortality were identified using multivariable Cox proportional hazards regression models. Prespecified covariates for the Cox models included age, sex, primary tumor site, ECOG performance status, residency, laboratory location, and biomarker status. All multivariable modeling and data visualizations were performed using Python (version 3.x). A p-value of < 0.05 was considered statistically significant.

### 2.5 Ethics approval

The project was categorized as a Quality Assurance (QA) initiative aimed at evaluating provincial care pathways; therefore, it was exempted from full Research Ethics Board (REB) review by the University of Saskatchewan. Operational approval was obtained from the Saskatchewan Cancer Agency (OA-UofS-E-Bio-039). As this was a retrospective review of de-identified administrative data for QA purposes, the requirement for informed consent was waived.

## 3 Results

### 3.1 Patient demographics

**Figure 1:**
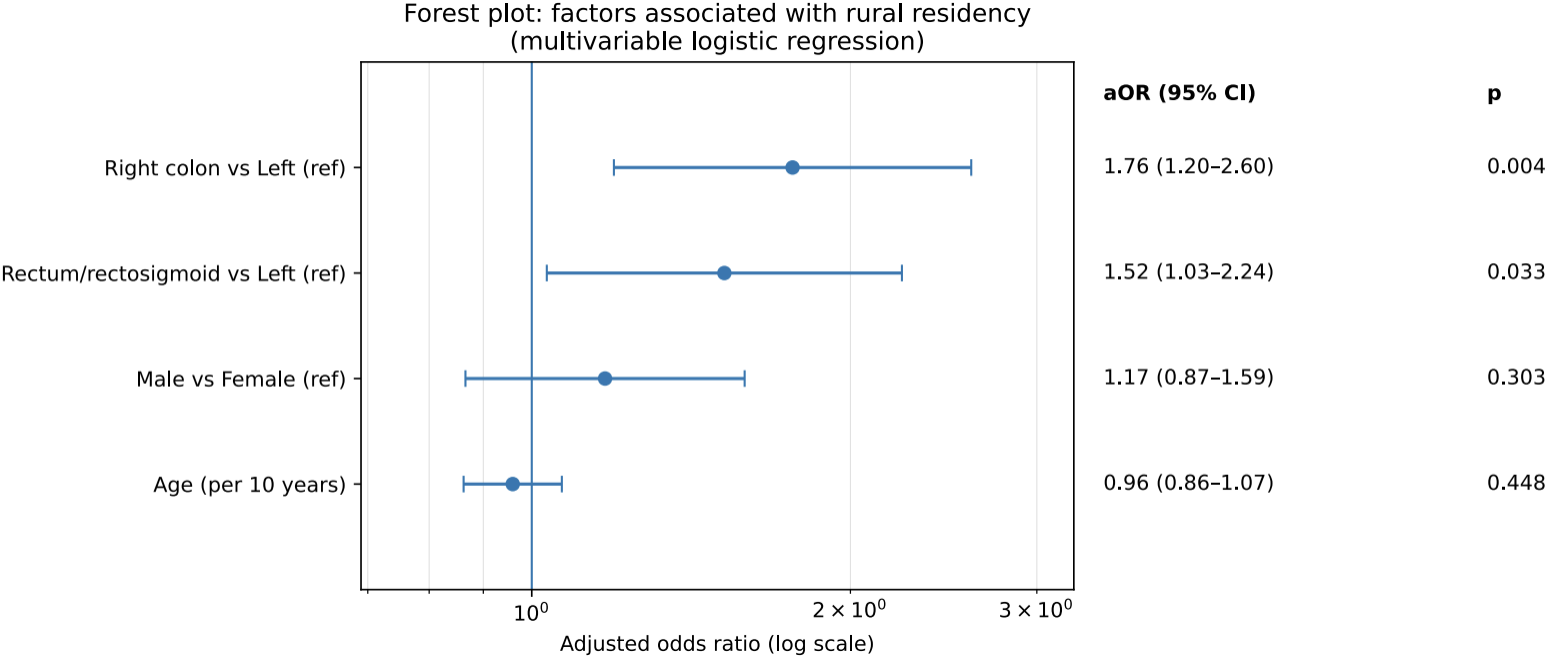
Points represent aORs, adjusted odds ratios and horizontal lines denote 95% confidence intervals from a logistic regression model with rural residency (yes/no) as the outcome. Covariates included age (modelled per 10-year increase), sex (male vs female), and primary tumour site grouped as right colon, left colon (reference), and rectum/rectosigmoid (rectum category includes rectosigmoid junction). The vertical line indicates aOR = 1.0 (no association). Numerical aORs with 95% CIs and two-sided p-values are shown in the right column.

Total of 818 patients were analyzed. The patients who were identified as rural (n=522) vs urban (n=296) patients had similar age (median 69 [IQR 61-78] vs 70 [IQR 59-82] years; p=0.388) and sex distributions (female 41.8% vs male 45.3%; p=0.368). Primary tumor site, grouped as right colon, left colon, and rectum (rectum including rectosigmoid junction), differed by residency (p=0.019): rural patients had a higher proportion of right-sided tumors (39.8% vs 33.1%), whereas urban patients had a higher proportion of left-sided tumors (19.0% vs 26.7%); rectal/rectosigmoid tumors were similar (35.4% vs 32.4%). A small fraction of cases was classified as other/unspecified (5.7% rural vs 7.8% urban). Diagnostic lab location also differed significantly by residency (p<0.001): 27.4% of rural residents were diagnosed in rural laboratories (143/522), while all urban residents were diagnosed in urban laboratories (296/296). After adjusting for age and sex, tumor site remained independently associated with residency. Compared with left-sided primaries, rural patients had higher odds of right-sided tumors (aOR 1.76, p=0.004) and rectal/rectosigmoid tumors (aOR 1.52, p=0.033), **Figure 1**. Age and sex were not independently associated with rural residency in this model **Table 2**.

When patients were dichotomized by age at diagnosis into Early-Onset CRC (EOCRC, <50 years; n=83) and Average/Late-Onset CRC (AOCRC, ≥50 years; n=735), the proportion of EOCRC was nearly identical between rural and urban patients (9.96% vs. 10.47% of rural and urban patients, respectively; Fisher’s exact p=0.811), confirming that early-onset disease is not differentially associated with rural residency in this cohort.

**Figure 1**.

### 3.2 Biomarker status, MSI/molecular markers

**Figure 2:**
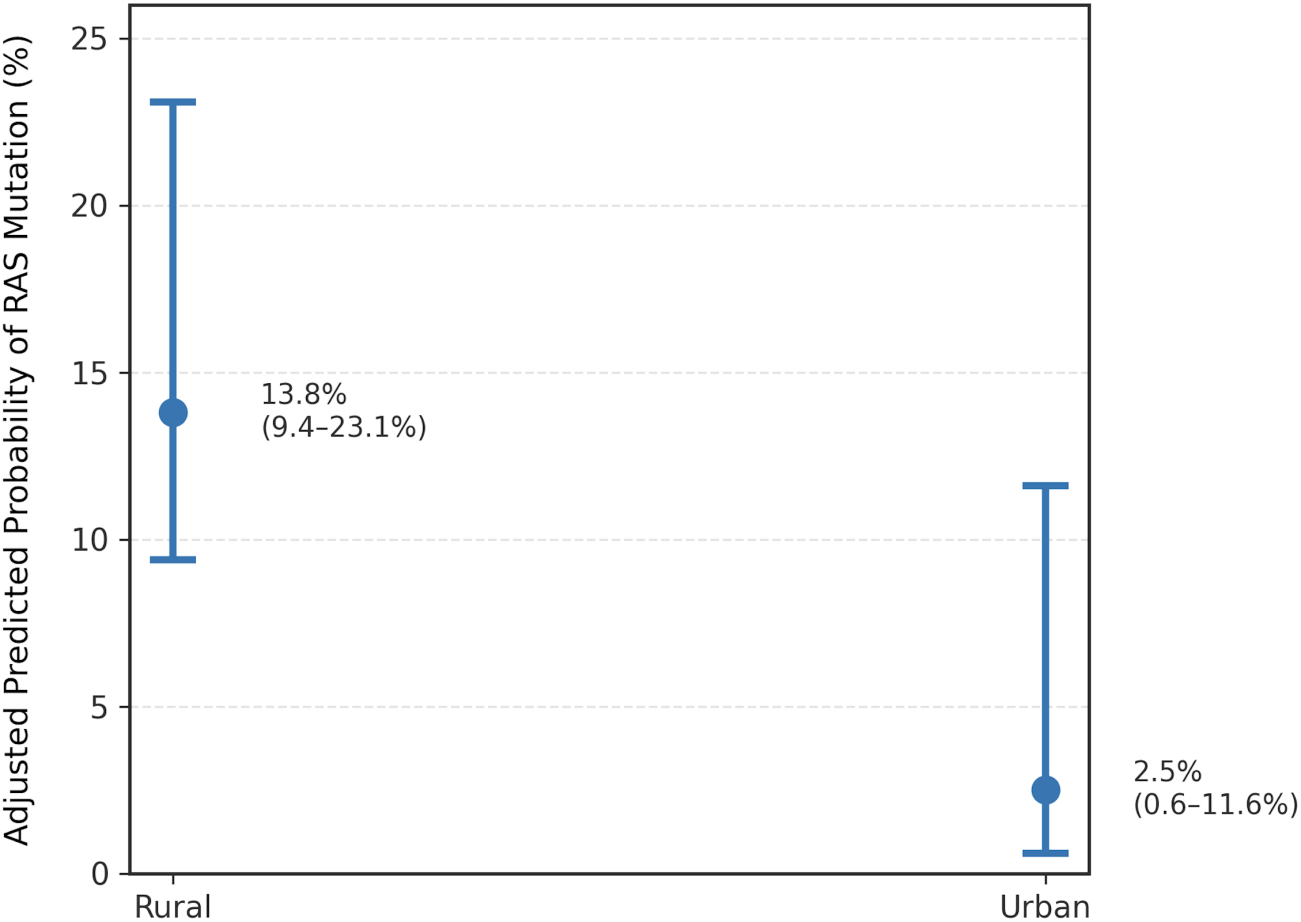
Adjusted predicted probability of RAS mutation by geographic residency. Points represent the marginal predicted probabilities (%) of harboring a RAS mutation derived from a Firth-penalized multivariable logistic regression model. Vertical whiskers denote the corresponding 95% confidence intervals. The adjusted probability is significantly higher among patients residing in rural areas (13.8%; 95% CI: 9.4%–23.1%) compared to those in urban environments (2.5%; 95% CI: 0.6%–11.6%).

Across biomarker-tested cases, the distribution of MSI-high and BRAF v600E status was similar between rural and urban residents. MSI-high was identified in 8.5% of rural tumors (21/247) versus 5.8% of urban tumors (8/139) (OR 0.66, 95% CI 0.28–1.53; Fisher p = 0.422), and BRAF v600E was present in 8.4% of rural tumors (10/119) versus 12.5% of urban tumors (8/64) (OR 1.56, 95% CI 0.58–4.17; Fisher p = 0.437).In contrast, RAS mutation status was defined as the presence of a mutation in either KRAS or NRAS, these data were available for only 22.0% of patients (180/818). Among this subset, the observed mutation prevalence was 8.3% (15/180). Given the sparse number of RAS-mutated events, we applied Firth penalized logistic regression to provide robust estimates; after adjusting for age, sex, year of diagnosis, and primary tumor site, urban residency remained associated with significantly lower odds of RAS mutation (aOR 0.15, 95% CI 0.03–0.79, p = 0.005) **Figure 2**; **Table 3**.

### 3.3 Treatment delays

Delays were calculated from the time of initial pathology diagnosis to the start of the treatment modality. Out of 818 patients, 591 underwent at least one surgical procedure. We excluded 207 cases in which the recorded surgical date preceded or coincided with the formal date of diagnosis, typically within ±1 day. These cases likely represent emergency presentations for acute abdominal symptoms in which malignancy was identified intraoperatively or only after surgery. They were therefore excluded to ensure that the analysis captured the interval between a known clinical diagnosis and a planned surgical intervention. Among the remaining 384 patients, 111 underwent primary tumor resection. The median time from diagnosis to surgery for rural-residing patients whose primary resection was performed in community hospitals was 34.9 days, compared with 33.5 days for urban-residing patients whose surgery was performed in urban referral centers. In addition, 53 patients underwent metastasectomy, all of which were performed in urban centers regardless of patient residence, and 220 underwent secondary resection for relief of obstruction. Overall, when all surgery types were considered together, the median time from diagnosis to surgery did not differ significantly between rural and urban residents (55 vs 52 days; p = 0.695). A non-significant trend toward longer chemotherapy delays was observed for rural residents (59 vs. 52 days; p = 0.072), with a similar pattern for immunotherapy (68 vs. 64 days; p = 0.16). However, when stratified by diagnostic laboratory location, patients whose initial pathology diagnosis occurred in rural pathology laboratories vs urban pathology laboratories experienced significantly longer delays from diagnosis to chemotherapy initiation (66.5 vs. 55 days; p = 0.011). A clinically relevant, though not statistically significant, delay was also noted for immunotherapy initiation in patients diagnosed at rural labs compared to urban labs (median 77.5 vs. 65 days; p = 0.114). Surgical timing remained comparable regardless of laboratory location (54.5 vs. 52 days; p = 0.131). Overall, these findings suggest that diagnostic pathways linked to rural laboratory locations are associated with later access to systemic therapies.

**Figure 3:**
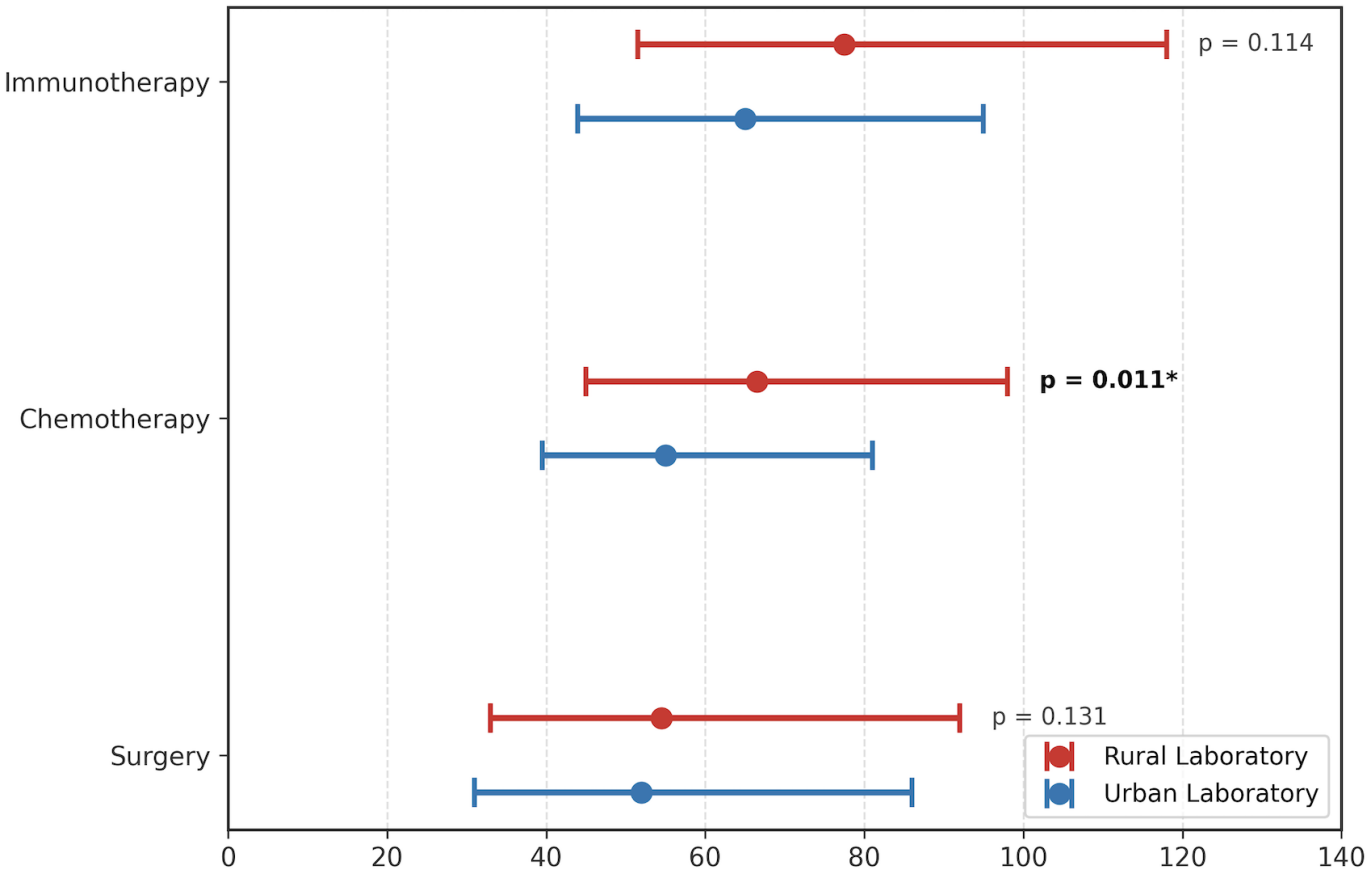
Time from diagnosis to treatment initiation by diagnosing lab location (rural vs urban), excluding perioperative diagnoses. Horizontal bars illustrate the turnaround time profiles (days) for critical therapeutic modalities (Immunotherapy, Chemotherapy, and Surgery) compared between rural laboratories (red lines) and urban laboratories (blue lines). Central points indicate the calculated model estimates and flanking error bars represent the 95% confidence intervals. A statistically significant difference in turnaround delay is observed exclusively within the chemotherapy biomarker cohort (*p* = 0.011*).

**Figure 3**; **Table 4**.

### 3.4 Survival

Kaplan–Meier analysis of overall survival (from date of diagnosis to death or last follow-up) showed no meaningful difference by patient residency. Among 818 patients, 522 rural and 296 urban, deaths occurred in 444 (85.1%) and 255 (86.1%), respectively. The median overall survival was 250 days for rural residents versus 270 days for urban residents, and the survival curves largely overlapped across follow-up; accordingly, the log-rank test was not significant(χ² = 0.083, p = 0.774). Estimated survival was also similar at clinically relevant landmarks (≈43.5% vs 44.3% at 1 year, and ≈9.4% vs 9.3% at 3 years for rural versus urban).

**Figure 4**.

**Figure 4:**
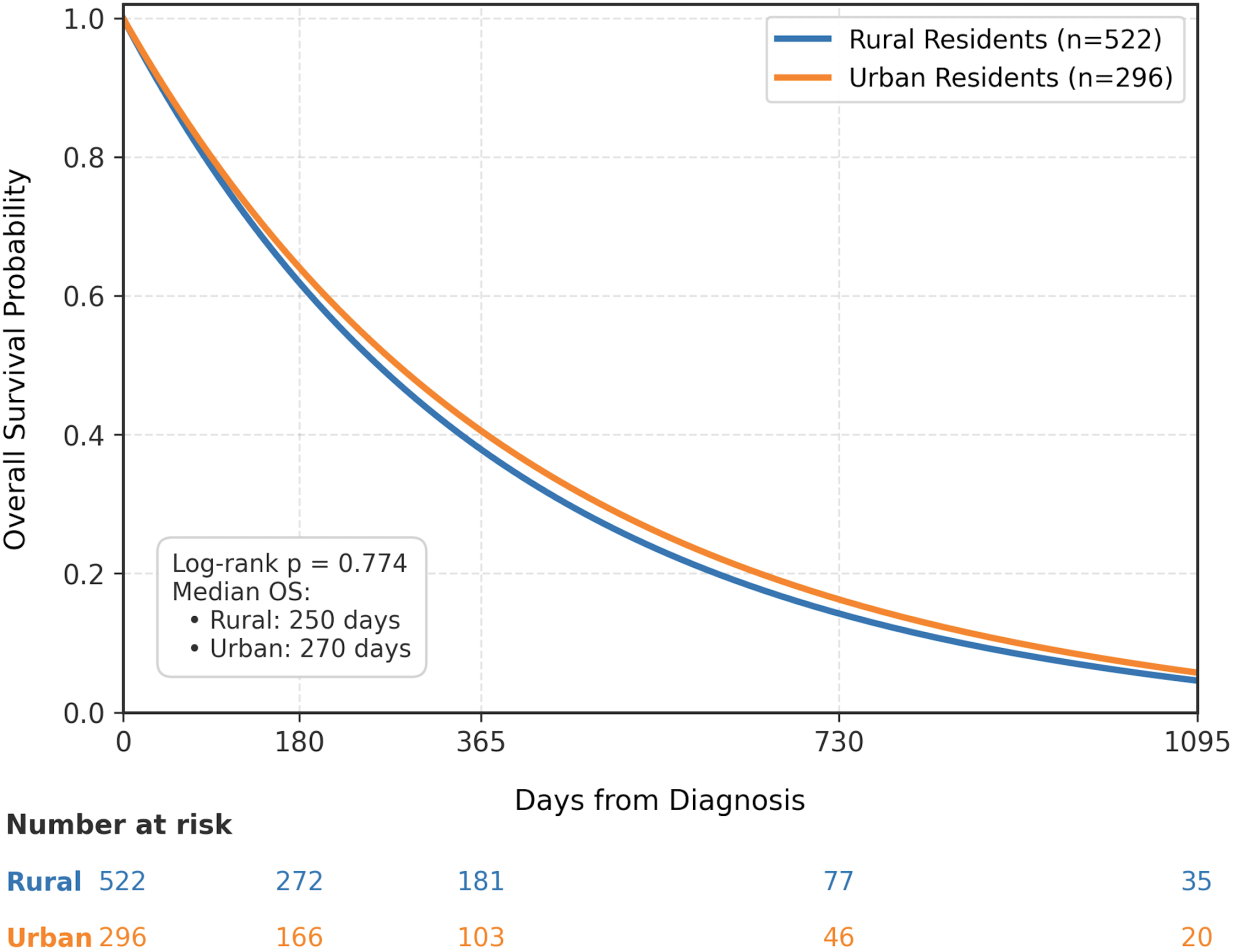
Kaplan–Meier overall survival by patient residency (rural vs urban). Survival curves demonstrate the probability of overall survival from the date of initial diagnosis to death or last follow-up for rural residents (n = 522, blue curve) versus urban residents (n = 296, orange curve). No statistically significant difference in overall survival was detected between the two cohorts (Log-rank *p* = 0.774). Median overall survival was 250 days for rural residents and 270 days for urban residents. The corresponding number of patients at risk over the 1,095-day follow-up interval is provided below the axis.

In the prespecified multivariable Cox proportional hazards model (overall survival from diagnosis to death/last follow-up; N = 818, deaths=699), older age and poorer performance status were the primary independent predictors of mortality. Each 10-year increase in age was associated with higher hazard of death (HR 1.15, 95% CI 1.08–1.22; p<0.001). ECOG demonstrated a strong dose–response effect (HR 1.57 per 1-point increase, 95% CI 1.43–1.73; p<0.001). After adjustment, residency (rural versus urban HR 1.03; p=0.754) and diagnosis in a rural lab (HR 1.15; p=0.209) were not significantly associated with survival. Similarly, tumor site (left colon or rectum/rectosigmoid vs right colon) was not associated with overall survival (all p>0.4), and no independent associations were observed for MSI status (MSI-high vs MSI-low HR 1.04; p=0.880), BRAF mutation (HR 1.09; p=0.769), or RAS mutation (HR 0.72; p=0.341). Receipt of chemotherapy was associated with a lower hazard of death (HR 0.67, 95% CI 0.55–0.82; p=0.0001), recognizing potential selection/timing effects. Site-specific metastases at diagnosis (bone, brain, liver, lung) were not independently associated with mortality in this model (all p>0.39).

**Figure 5**.

**Figure 5:**
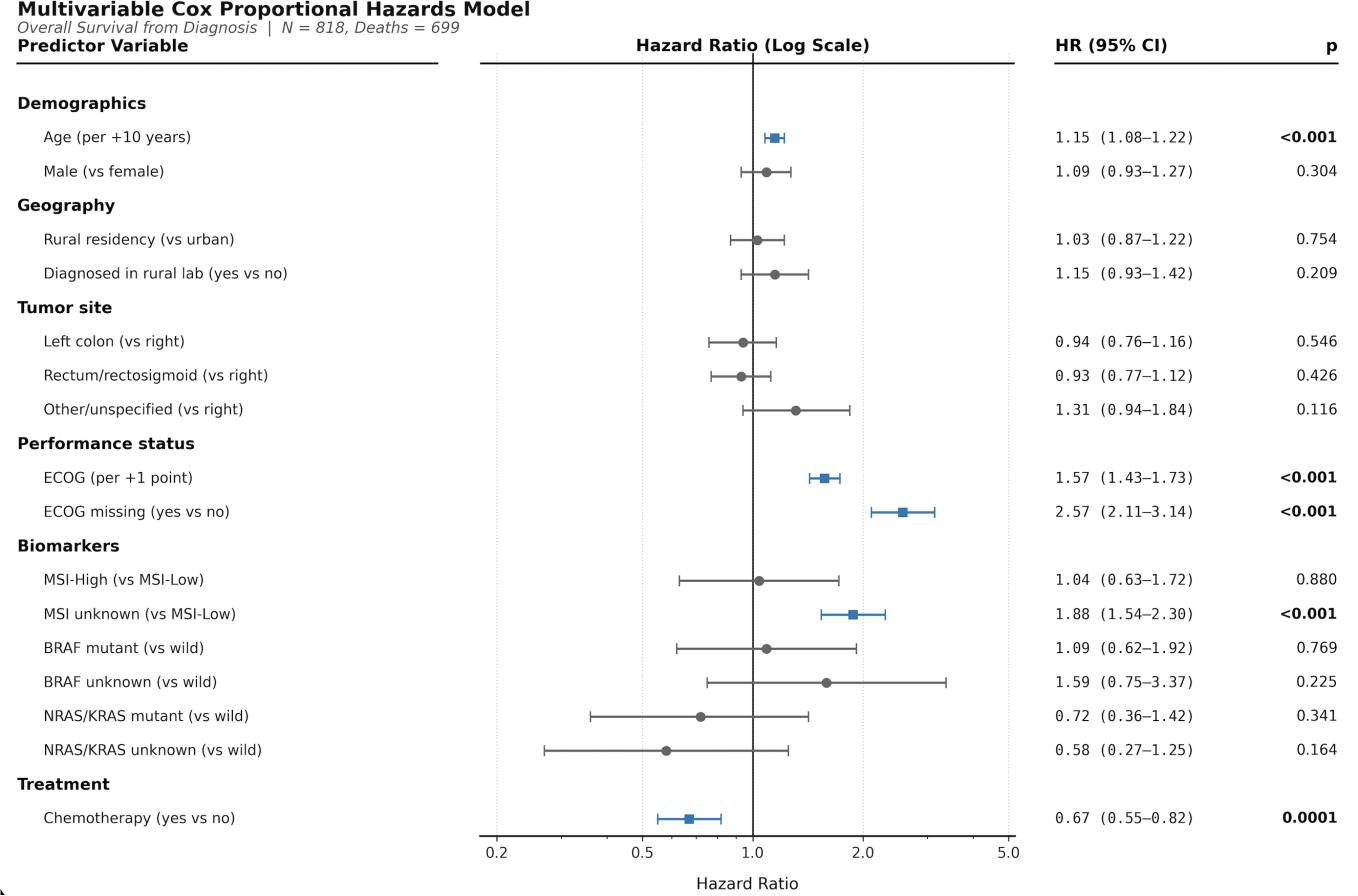
Multivariable Cox proportional hazards model for overall survival. Points represent hazard ratios (HRs) with horizontal lines denoting 95% confidence intervals (CIs) from a multivariable model adjusting for patient demographics, geographic metrics, tumor location, performance status, molecular biomarkers, and treatment status. Square blue markers highlight statistically significant independent predictors of mortality (p < 0.05), while grey circular markers denote non-significant associations. Numerical hazard ratios, 95% CIs, and two-sided p-values are provided on the right.

## 4 Discussion

### 4.1 Regional and diagnostic pathway differences in treatment time

In this contemporary, population-based cohort of patients with stage IV colorectal cancer (CRC) in Saskatchewan, rural–urban inequities were not uniform across the care continuum.

The absence of a regional difference in time-to-surgery is notable given prior Canadian data reporting longer surgical wait times in rural settings (14). A plausible explanation is case-mix: this cohort was restricted to stage IV disease, where surgery is not the standard of care and often undertaken for urgent indications such as obstruction or bleeding and therefore prioritized similarly regardless of geography. More broadly, Canadian studies suggest that delays in cancer care frequently arise upstream, during diagnostic workup and care coordination, rather than during the surgical episode itself (15).

Delays in this cohort were concentrated in systemic therapy initiation. While median times to chemotherapy and immunotherapy did not differ significantly by patient residence, patients diagnosed through rural laboratories experienced longer times to chemotherapy initiation, with similar trends observed for immunotherapy. This pattern aligns with evidence that access to ongoing oncology care is influenced by system-level factors, including diagnostic workflows, referral pathways, and coordination across sites, particularly when care is delivered outside tertiary centers (10),^4^. Although Saskatchewan’s Community Oncology Program of Saskatchewan aims to provide oncology services closer to home ^5^, additional coordination steps related to pathology reporting, treatment authorization, and infusion capacity may extend timelines for patients diagnosed in non-urban settings (16).

The observed delays in immunotherapy initiation are clinically relevant given the biomarker-defined nature of immunotherapy use in mCRC. Delays can accumulate across multiple steps, including specimen retrieval, MSI/MMR testing and reporting, oncology consultation, and treatment scheduling. Inefficiencies at any point may delay treatment initiation and potentially limit therapeutic benefit^6^.

Our results suggest that in mCRC, the “key bottleneck” may be the efficiency of centralized testing and referral mechanisms embedded in the diagnostic laboratory network. Taken together, these findings raise an important policy question: whether current centralized referral models for biomarker testing are optimized for timely mCRC decision-making, or whether targeted solutions—such as streamlined specimen routing and accessioning, reflex biomarker testing protocols, integrated reporting, and/or strengthening regional.

### 4.2 RAS mutations

Two molecular findings merit cautious interpretation and, importantly, point to test-access and treatment-era effects rather than straightforward biologic conclusions. First, “RAS mutations appeared more frequent among rural residents and remained associated after adjustment; however, this finding warrants cautious interpretation given the unexpectedly low overall RAS mutation prevalence in our cohort (15/180; 8.3%). Of note, RAS data were available for 22.0% of the full cohort (180/818), and the overall mutation prevalence was lower than expected given that RAS alterations are typically observed in approximately half of tumors in Canadian mCRC populations (16). These data raise the possibility that incomplete or selective molecular testing, missingness, or other residual selection mechanisms could contribute to the observed rural association in our cohort. Saskatchewan also has a high proportion of Indigenous peoples (17% of the provincial population)^6^, and emerging genomic literature suggests that genetic ancestry can correlate with colorectal cancer molecular phenotypes (17) nonetheless, Indigenous-specific data on RAS mutation prevalence in Canadian mCRCremain limited. Confirmation in larger cohorts with standardized, expanded KRAS/NRAS testing and more complete capture of relevant sociodemographic variables is therefore needed.

Second, the lack of association between BRAF mutation or MSI-high status and overall survival contrasts with the established prognostic/predictive literature in metastatic disease and likely reflects a combination of treatment era, incomplete biomarker ascertainment, and limited subgroup power. BRAFV600E defines an aggressive mCRC subtype and has repeatedly been associated with inferior survival in meta-analyses and pooled trial datasets (18). For MSI-H/dMMR mCRC, immune checkpoint inhibition has become foundational for better outcome (19). Together, these considerations support interpreting the BRAF/MSI survival findings primarily as signals of data completeness and therapy delivery (including timing and receipt of targeted/ICI regimens), rather than evidence against their established clinical relevance. Contemporary guidelines emphasize timely biomarker testing as a prerequisite for appropriate subtype-directed therapy, reinforcing the need for robust, equitable testing pathways when evaluating outcomes by molecular subgroup.

### 4.3 Overall survival

The median overall survival observed in our cohort (250–270 days) may appear lower than survival figures often cited for metastatic colorectal cancer in the contemporary literature. However, those estimates commonly derive from treated or trial-enriched cohorts and are not directly comparable to a population-based, all-comers stage IV cohort such as ours. At the Canadian population level, stage IV colorectal cancer continues to carry a poor prognosis, with five-year net survival estimated at 12% for colon cancer and 13% for rectal cancer (7). By contrast, in a large Ontario population-based study restricted to patients who actually received first-line palliative chemotherapy for unresectable locally advanced or metastatic colorectal cancer, median overall survival was 17.1 months, and the authors noted that even this real-world treated cohort had outcomes inferior to pivotal clinical trials (20). Accordingly, the shorter survival observed in our study likely reflects the inclusion of the full provincial stage IV population, including older, frailer, and untreated patients, rather than inconsistency with Canadian population-level data.

## 5 Strengths, limitations, and conclusions

This study captures a contemporary, province-wide cohort of patients with stage IV CRC in the era of biomarker-informed care and integrates clinical, molecular, treatment timing, and survival data. Considering both patient residence and diagnostic laboratory location offers insight into how health system structure and diagnostic pathways may influence access to treatment.

The retrospective design and use of registry data introduce potential limitations, including incomplete or imprecise date fields. in addition, the limitations inherent to a retrospective study and the broad time frame includes variability in treatment regimens especially using immunotherapy. Data on pre-diagnostic delays, referral timelines, oncology consultation timing, biomarker turnaround, and key clinical and social determinants were available for some patients but not uniformly across the cohort, limiting full adjustment. Molecular analyses were restricted to tested cases, and some subgroup analyses may be underpowered. Additionally, information on whether metastatic disease was synchronous or metachronous at diagnosis was not available through the provincial registry system, which precludes subgroup analyses based on timing of metastasis. A proportion of cases were coded as “COLON NOS” in the registry and could not be mapped to a specific anatomical site without individual record review; this accounts for the small “other/unspecified” category in **Table 1**. Future studies focusing on a more recent, standardized treatment era—such as the post-2020 period when biological agents and metastasectomy were more consistently implemented provincially—would enable a more rigorous examination of rural–urban differences in treatment intensity, access to biologics, and surgical candidacy, particularly among elderly patients.

**Table 1:** 3.1 Patient demographics.

Characteristic	Rural (n=522)	Urban (n=296)	p-value
Age, years (median, IQR)	69 (61–78)	70 (59–82)	0.388
Sex			0.368
└ Female	218 (41.8%)	134 (45.3%)	
└ Male	304 (58.2%)	162 (54.7%)	
Tumor site			0.019
└ Right colon	208 (39.8%)	98 (33.1%)	
└ Left colon	99 (19.0%)	79 (26.7%)	
└ Rectum/rectosigmoid	185 (35.4%)	96 (32.4%)	
└ Other/unspecified	30 (5.7%)	23 (7.8%)	
Diagnosing lab location			<0.001
└ Rural lab	143 (27.4%)	0 (0.0%)	
└ Urban lab	379 (72.6%)	296 (100.0%)	
Age-onset group			0.811
└ EOCRC (<50 years)	52 (9.96%)	31 (10.47%)	
└ AOCRC (≥50 years)	470 (90.04%)	265 (89.53%)	

In summary, overall survival and access to surgery for stage IV CRC appear similar across rural and urban populations in Saskatchewan. However, delays in systemic therapy initiation; particularly among patients diagnosed through rural laboratories represent a potentially actionable gap in the care pathway.

Interventions focused on streamlining diagnostic-to-oncology workflows and improving coordination between diagnostic sites and cancer centers may help reduce disparities in contemporary mCRC care.

**Table 2:** Multivariable logistic regression of factors associated with rural residency.

Predictor	Adjusted OR (aOR)	95% CI	p-value
Right colon vs Left (ref)	1.76	1.20–2.60	0.004
Rectum/rectosigmoid vs Left (ref)	1.52	1.03–2.24	0.033
Age (per 10 years)	0.96	0.86–1.07	0.448
Male vs Female (ref)	1.17	0.87–1.59	0.303

**Table 3:** Biomarker status by patient place of residence. Denominators reflect only patients with available test results for each respective biomarker.

Outcome	Rural	Urban	OR (urban vs rural)	Fisher p
MSI-high	21/247 (8.5%)	8/139 (5.8%)	0.66 (0.28–1.53)	0.422
BRAF v600E	10/119 (8.4%)	8/64 (12.5%)	1.56 (0.58–4.17)	0.437
RAS mutated	13/116 (11.2%)	1/63 (1.6%)	0.13 (0.02–1.00)	0.021

**Table 4:** Treatment delays.

Comparison	Surgery (n; median)	Chemotherapy (n; median)	Immunotherapy (n; median)
Patient Residency:	247; 55	268; 59	91; 68
Rural			
Patient Residency:	137; 52	153; 52	67; 64
Urban			
p-value (Mann– Whitney)	0.695	0.072	0.16
Patients diagnosed in rural lab	58; 54.5	68; 66.5	24; 77.5
Patients diagnosed in urban lab	326; 52	353; 55	134; 65
p-value (Mann- Whitney)	0.131	0.011	0.114

## Data Availability

The raw data supporting the conclusions of this article will be made available by the authors, without undue reservation.
